# Effect of iterative reconstruction and temporal averaging on contour sharpness in dynamic myocardial CT perfusion: Sub-analysis of the prospective 4D CT perfusion pilot study

**DOI:** 10.1371/journal.pone.0205922

**Published:** 2018-10-16

**Authors:** Sarah Feger, Carsten Kendziorra, Steffen Lukas, Ahmed Shaban, Björn Bokelmann, Elke Zimmermann, Matthias Rief, Marc Dewey

**Affiliations:** Department of Radiology, Charité—Universitätsmedizin Berlin, Berlin, Germany; Chongqing University, CHINA

## Abstract

**Purpose:**

Myocardial computed tomography perfusion (CTP) allows the assessment of the functional relevance of coronary artery stenosis. This study investigates to what extent the contour sharpness of sequences acquired by dynamic myocardial CTP is influenced by the following noise reduction methods: temporal averaging and adaptive iterative dose reduction 3D (AIDR 3D).

**Materials and methods:**

Dynamic myocardial CT perfusion was conducted in 29 patients at a dose level of 9.5±2.0 mSv and was reconstructed with both filtered back projection (FBP) and strong levels of AIDR 3D. Temporal averaging to reduce noise was performed as a post-processing step by combining two, three, four, six and eight original consecutive 3D datasets. We evaluated the contour sharpness at four distinct edges of the left-ventricular myocardium based on two different approaches: the distance between 25% and 75% of the maximal grey value (d) and the slope in the contour (m).

**Results:**

Iterative reconstruction reduced contour sharpness: both measures of contour sharpness performed better for FBP than for AIDR 3D (d = 1.7±0.4 mm versus 2.0±0.5 mm, p>0.059 at all edges; m = 255.9±123.9 HU/mm versus 160.6±123.5 HU/mm; p<0.023 for all edges). Increasing levels of temporal averaging degraded contour sharpness. When FBP reconstruction was applied, contour sharpness was best without temporal averaging (d = 1.7±0.4 mm, m = 255.9±123.9 HU/mm) and poorest for the strongest levels of temporal averaging (d = 2.1±0.3 mm, m = 142.2±104.9 HU/mm; comparison between lowest and highest temporal averaging level: for d p>0.052 at all edges and for m p<0.001 at all edges).

**Conclusion:**

The use of both temporal averaging and iterative reconstruction degrades objective contour sharpness parameters of dynamic myocardial CTP. Thus, further advances in image processing are needed to optimise contour sharpness of 4D myocardial CTP.

## Introduction

While non-invasive computed tomography coronary angiography (CTA) is highly accurate in diagnosing coronary artery stenosis [1, 2] compared with conventional coronary angiography (CCA), it is limited in the assessment of the haemodynamic relevance of coronary stenosis [3]. This is especially relevant in patients with intermediate coronary diameter stenosis of 30–70% [3] or heavily calcified plaques and in patients with coronary stents [4], which might reduce the evaluability of the corresponding coronary segment due to artefacts resulting from the stent strut.

Myocardial CT perfusion (CTP) is a promising approach to detect myocardial ischaemia as a predictor of the functional relevance of a coronary stenosis diagnosed during CTA [5, 6]. Compared with single-photon emission CT, myocardial perfusion imaging (SPECT-MPI) or stress magnetic resonance imaging (MRI), which are commonly used diagnostic tests in clinical routine, CTP has a very high diagnostic accuracy [7–9]. A relevant advantage of myocardial CTP is the possibility to perform anatomic and functional assessment (CTA and CTP) in a single session [10], which further improves the diagnostic correctness as compared with CTP alone [11, 12]. In general, CTP can be performed as static myocardial CTP with data acquisition at one single time point [13] or as dynamic four-dimensional myocardial CTP (4D-CTP) [14]. Enabling the acquisition of images at different points in time, this approach allows the quantitative analysis of the absolute myocardial blood flow. Additionally, time attenuation curves can be generated during the first pass, the arterial phase and the microcirculation phase of contrast agent enhancement [15]. Currently, only 4D CTP allows estimation of the absolute myocardial blood flow from the respective input and output function [16].

Despite the huge potential of 4D CTP there are important challenges that have to be addressed. Multiple acquisitions lead to potentially high radiation exposure [15], while low-dose acquisition results in overall higher noise levels. Additionally, there are exam intrinsic artifacts from highly attenuating tracer (beam hardening) and cardiac motion and deformation.

Temporal averaging of multiple datasets and iterative reconstruction techniques as means of noise reduction reduce beam hardening artefacts but may lead to poorer image sharpness from blurred edges. Two feasibility studies of temporal averaging have been published so far [17, 18] and this is one of the largest whole-heart CT myocardial perfusion dataset studies to date.

The purpose of this study was to objectively analyse the effects of temporal averaging and iterative reconstruction on contour sharpness in myocardial 4D CTP.

## Materials and methods

### Study design

**Fig 1:**
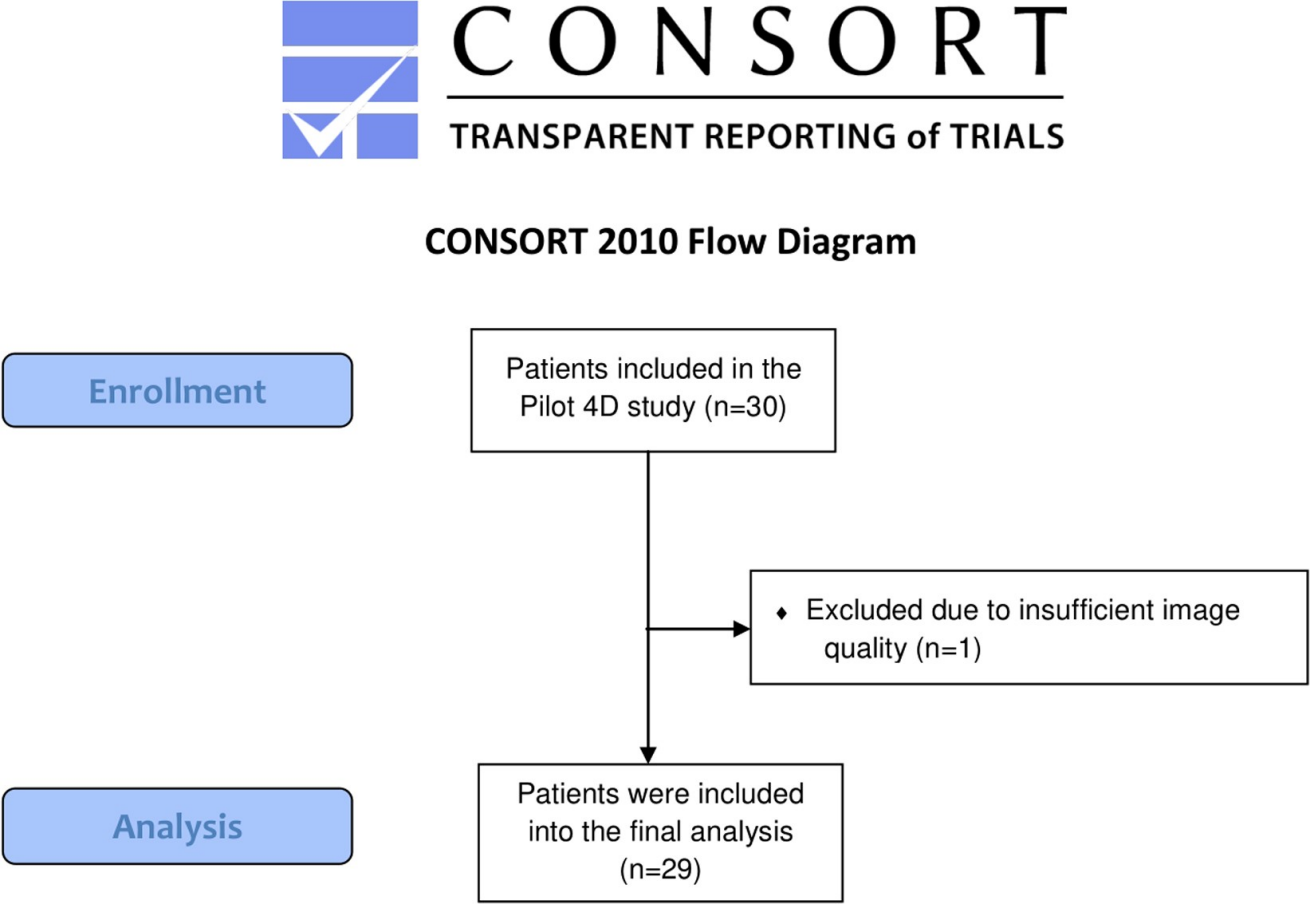
CONSORT flow diagramme. Flow diagramme of the 4D CTP Pilot Image Quality sub-study. Thirty patients were eligible and included in the pilot study. Because of the intra-individual study design, this flow chart does not include a randomisation process. See the main study for further details with regard to inclusion and exclusion criteria, patient recruitment and follow-up (Feger et al. IJCVI 2016). One patient was excluded from the final analysis due to insufficient general image quality of CTA and CTP, leaving 29 patients for the final analysis.

This is the contour sharpness sub-study of the prospective 4D CT perfusion pilot study [18]. A detailed description of in- and exclusion criteria, patient preparation and CT protocol has already been published with the main study. Altogether, we included 29 patients (Fig 1) who underwent both, cardiac CTA and dynamic myocardial CTP, on a 320-row CT scanner (Aquilion ONE, Toshiba Medical Systems, Otawara, Japan). 4D CTP was performed after adenosine administration, and patients received intravenous contrast agent for both the CTA and CTP examination. All patients were referred for cardiac CT with a clinical indication. We included patients with suspected or known coronary artery disease (CAD) who were at least 40 years of age. In the following cases, the patients were not eligible: any coronary intervention during the last three months, history of coronary artery bypass grafting, creatinine >2.0 mg/dl, no sinus rhythm, heart failure (NYHA III or IV), moderate to severe aortic stenosis, severe chronic obstructive pulmonary disease, pregnancy, sildenafil intake in the last 48 hours, chronic dipyridamol treatment, inability to hold the breath for 15 s, pre-existing severe hypotension, atrioventricular block of grade 2 or 3, sick sinus syndrome, and unstable angina pectoris. Our Institutional Review Board approved this prospective pilot study, and all patients gave written informed consent (Ethikausschuss 1 at Campus Charité–Mitte; Chairman Prof. Dr. R. Uebelhack; EA1/251/11). The study was performed according to the principles stated in the Declaration of Helsinki.

### Patient preparation

To lower the heart rate and variability, patients with a heart rate of >60 bpm received beta blockers before the CT examination (n = 14 only oral beta blockers [atenolol, Tenormin 50, Astra-Zeneca] and n = 6 oral and IV beta blockade [esmolol, Brevibloc, Baxter]). Another three patients were administered 5 mg (1 patient) or 10 mg (2 patients) of ivabradin (Procoralan, Servier). Immediately before the CT scan all patients received sublingual nitroglycerine (1.2 mg). The patients were asked in advance not to ingest any xanthine-containing food and beverages for at least 12 hours before adenosine injection. The administration of xanthine-containing medicine (theophylline) was avoided for a 24-hour interval before the adenosine injection due to possible antagonistic effects of xanthine, which could degrade the effect of adenosine.

### Coronary CTA and dynamic myocardial 4D CTP

Coronary artery calcium scoring (CACS) was performed without injection of contrast agent at 100 kV and 140 mA, covering 14 cm of the heart in z-direction. The CTA was planned based on the position of the coronary arteries on CACS plus 15 mm in each direction of the z-axis. The tube voltage was 120 kV for patients with a body mass index (BMI) < 36 und 135 kV for BMI ≥ 36. See the main manuscript for further details. For both, the CTA and CTP, the patients received non-ionic contrast agent (iobitridol, 350 mg of iodine per millilitre, Xentix 350, Guerbet, France) at a flow rate of 7 ml/s through an IV line in the right antecubital vein. For CTA and CTP, the patients were administered 58 ml and 42 ml of contrast agent, respectively, followed by a saline flush of 80 ml. CTP always followed the CTA and was initiated after continuous infusion of adenosine for 3 min.

The CTP coverage in z-direction was calculated from the extent of the myocardium in z-direction on the CACS scan plus 10 mm in each direction in order to not miss parts of the myocardium during dynamic CTP, or plus 15 mm in the case of a large positional deviation in z-direction between CACS before CTA and CTP. To determine the CTP start point after contrast agent injection, the arterial input function of the CTA SureStart was used. In each case, scanning was performed at 70–80% of the RR interval. The dynamic CTP with one acquisition every heart beat was followed by three single late phases 10, 20 and 35 s after the dynamic scan. In order to cover 20 heart beats, the total scan time of the dynamic phase was adjusted to the individual heart rate. CTP was initiated after continuous adenosine infusion for 3 min. Images were acquired with a gantry rotation time of 350 ms, and we used a tube voltage of 100 kV and tube current of 140 mA for patients with BMI ≤ 30 and 200 mA for BMI > 30. The vasodilatory effective adenosine was adapted to the body weight (140 μg/kg/min) and administered continuously over a maximum period of 6 min by an IV infusion through the left cubital vein using a perfusion system.

### Image quality evaluation

#### CT image reconstruction

We performed the CT image reconstruction by using an imaging matrix of 512 x 512 pixels covering a field of view (FOV) of 180 mm in axial direction. The reconstructions were performed in 5% intervals of the available RR interval in all patients, and additionally of the visually chosen best phase. Cardiac volumes were reconstructed using two methods: filtered back projection (FBP) and Adaptive Iterative Dose Reduction 3D (AIDR-3D) [11]. For the latter, the strong level was chosen, as this has been shown to yield superior objective image quality parameters compared to mild and standard levels [19]. Slice thickness was 0.5 mm with 0.25 mm slice increment.

#### Temporal averaging

Assuming little cardiac motion and deformation, we applied temporal averaging of several consecutive 3D datasets for noise reduction. The cardiac phase detection algorithm [20] was used to select a reference point of time t for each patient with high contrast in the left ventricle and low contrast in the right ventricle, since this is the time point with the strongest contrast in the region of interest but also the phase with the most severe beam hardening. Next, we averaged a varying number of the neighbouring volumes t_i_ centred around the reference time point t. The averaging level N (N = 1, 2, 3, 4, 6, 8) designates the number of additions during the temporal averaging. The averaged volumes are the arithmetic means of the input volumes in the time domain. The intensity I of a voxel at position x and time t is obtained from N equally weighted consecutive voxel intensities according to the formula

$$I_{N\;combined}(\vec{x},t)=\frac{1}{N}\sum_{i=1}^{N}I(\vec{x},t_{i})$$

We combined the different original 4D datasets from consecutive heart beats into one new 3D dataset to test the different combination levels. This resulted in six temporally averaged 3D volumes with the different levels of temporal averaging for each of the 29 patients and a total of 174 volumetric averaged datasets for all patients. This process was performed before reading as a post-processing step. See the main manuscript for further details. Only the FBP 4D CTP dataset was taken into account for the temporal averaging analysis in order not to overlay AIDR3D-instrinsic spatially diffusive smoothing with the averaging along the time dimension, which would result in overly blurred images. For the same reason, the comparison between FBP and AIDR 3D was performed without applying temporal averaging.

#### Contour sharpness analysis

**Fig 2:**
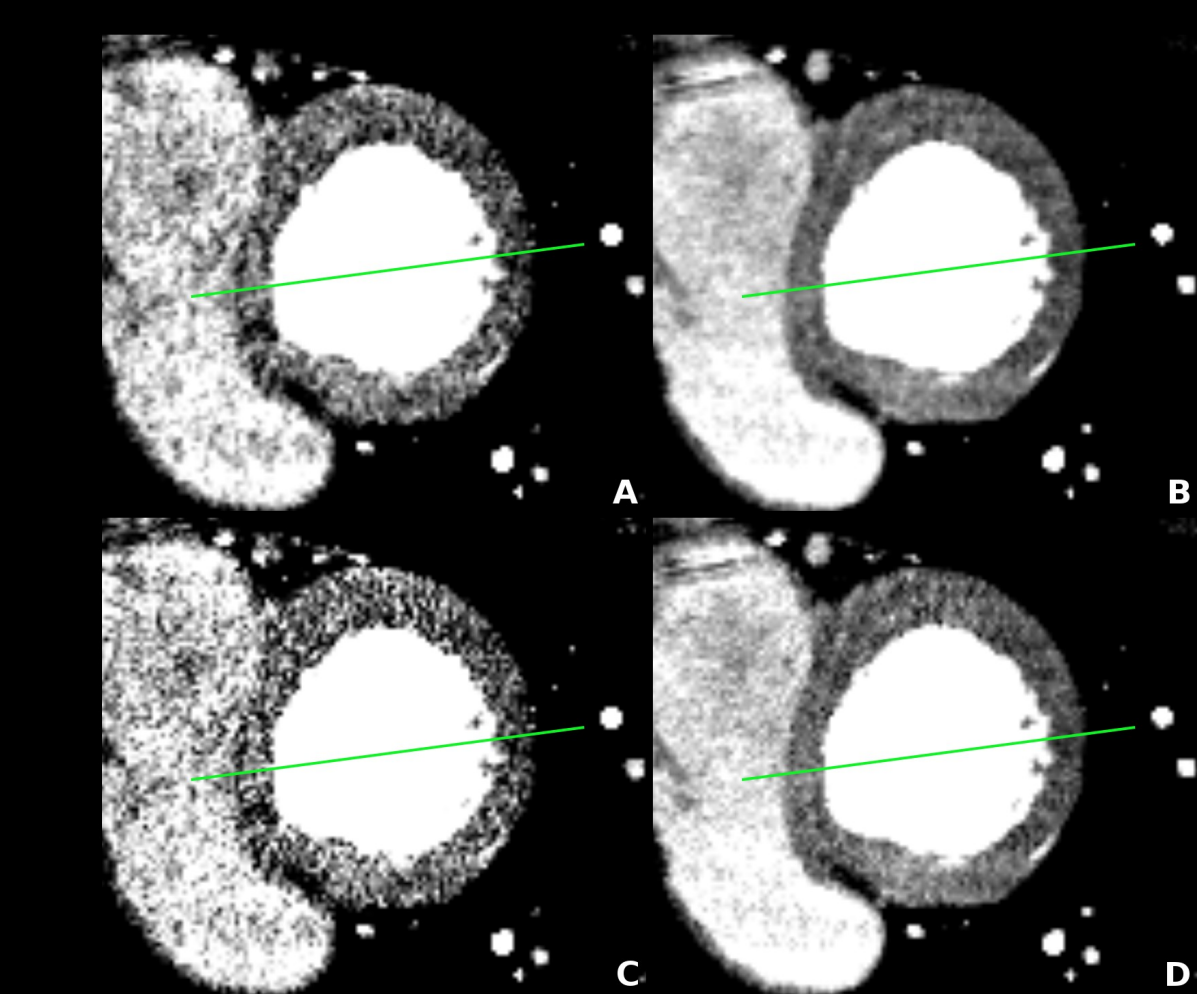
Contour sharpness measurement. Measurement of the contour sharpness was performed by placing a straight line above the heart connecting the myocardium of the left and the right ventricle, taking great care to avoid the inclusion of any perfusion defects or blooming artefacts into the measurement. Thus, we included 4 different representative localisations to cover the myocardium localised between: 1) the right ventricle and the septal myocardium, 2) the septal myocardium and left ventricle, 3) the left ventricle and lateral left ventricular myocardium and 4) the lateral left ventricular myocardium and surrounding epicardial tissue. To ensure exactly the same localisation for the different reconstructions and temporal averaging levels, the geometric coordinates of the measuring line were used for the different combinations: AIDR 3D without temporal averaging (A), AIDR 3D with temporal averaging (B), FBP without temporal averaging (C) and FBP with temporal averaging (D).

All 174 4D CTP datasets were analysed using the CardiacPerfusion software package (https://github.com/CardiacImagingCharite/CardiacPerfusion). For the analysis of the contour sharpness, a straight line was placed above the heart, displayed as 4-chamber view, to connect the myocardium of the left and the right ventricle, taking care to avoid the inclusion of any perfusion defects or blooming artefacts into the measurement. Thus, we integrated 4 different representative edge localisations of the myocardium between: 1) the right ventricle and the septal myocardium, 2) the septal myocardium and left ventricle, 3) the left ventricle and lateral left ventricular myocardium and 4) the lateral left ventricular myocardium and surrounding epicardial tissue (Fig 2).

We used two different measures to objectively evaluate the contour sharpness: first, the distance between the points of 25% and 75% of the maximal grey value along the edge section to exclude outliers from the analysis and, second, the slope m defined as the difference between 75% and 25% of grey values in the contour along the edge section divided by the distance calculated before.

$$d=\vec{x}(75\%\;of\;maximal\;grey\;value)-\vec{x}(25\%\;of\;maximal\;grey\;value)$$

m=(75%−25%ofmaximalgreyvalue)/d

**Fig 3:**
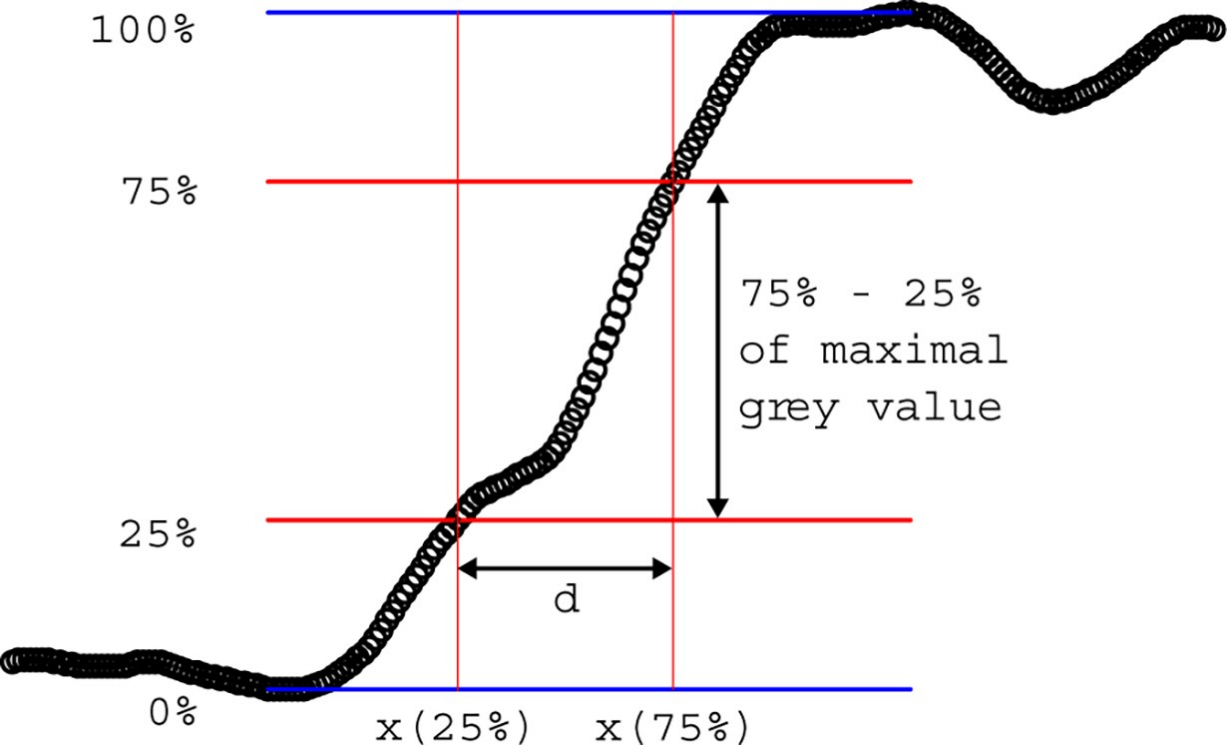
Contour sharpness parameters. Illustration of the measurement of the two different contour sharpness parameters. We used two different parameters to objectively evaluate the contour sharpness: the distance between 25% and 75% of the maximal grey value and the slope defined as the difference between 75% and 25% of the maximal grey value in the contour divided by the distance calculated before. d (Distance) = x(75% of the maximal grey value)–(25% of the maximal grey value) m (Slope) = (75% of maximal grey value– 25% maximal grey value)/d.

Hence, a sharp contour would be characterised by a small distance between the points of 25% and 75% of the maximal grey value (first parameter d) and high value of the slope in the contour (second parameter m; Fig 3).

### Statistical analysis

Values are given as arithmetic mean (standard deviation) if not mentioned otherwise. We performed the statistical analysis using SPSS version 20. The analyses were performed as intraindividual comparisons. First, we used the Shapiro-Wilk test to check normal distribution. If the values were normally distributed, we used ANOVA as overall test for repeated measurements for the comparison of the different temporal averaging levels, and the t-test for dependent values was used for the respective single comparisons. If normal distribution was refused, we used the Friedman test as overall test and the Wilcoxon signed-rank test for the single comparisons. Since we included only two different reconstructions into this analysis (FBP and AIDR 3D), overall testing was not necessary for the comparison of the reconstructions. The normally used p value of <0.05 was adapted here yielding <0.003 according to Bonferroni corrections for the fifteen possible comparisons of the five temporal averaging levels: 1–2, 1–3, 1–4, 1–6, 1–8, 2–3, 2–4, 2–6, 2–8, 3–4, 3–6, 3–8, 4–6, 4–8, 6–8. For the comparison of the two reconstructions, a p value of <0.05 was considered to indicate statistically significant differences.

## Results

### Patient characteristics

The 29 included patients had a mean age of 64±11 years, and there was a male-to-female ratio of 9:1 (Table 1). More than 80% of the patients had any type of chest pain with approximately one third suffering from typical angina pectoris. Almost two third of the patients had known CAD with prior myocardial infarction. All examinations were successfully performed, resulting in 174 4D CTP temporally averaged datasets.

**Table 1:** Patient characteristics.

Feature			
*Age*		63.7	±11.4 years
*Sex*			
	Female	3	(10%)
	Male	26	(90%)
*BMI*		26.5	±6.2 kg/m^2^
*Premedication*			
	Atenolol	20	(69%)
	Dose	97.5	±35.3 mg
	Ivabradin	3	(10%)
	Dose	6.7	±2.9 mg
	I.v. beta blockers	6	(21%)
	Dose	350.0	±207.4 mg
*Angina*			
	Typical	11	(38%)
	Atypical	5	(17%)
	Thoracic pain	9	(31%)
	No pain	4	(14%)
	Dyspnoea	10	(34%)
*CCS*			
	0	12	(41%)
	I	6	(21%)
	II	9	(31%)
	III	2	(7%)
*Cardiac insufficiency*		1	(3%)
*Previous myocardial infarction*		18	(62%)
*CTA*		358.6	±59.9 mA
		119.8	±4.7 kV
*CTP*		148.3	±21.1 mA
		103.4	±7.7 kV

The analysis included 29 patients with a mean age of 63.7±11.4 years. The majority of patients were male (ratio of 9:1). One third of the patients suffered from typical angina pectoris, while only 20% of the patients presented without any chest pain.

More than half of the patients (62%) had known CAD with a prior myocardial infarction.

Values are given as mean ± standard deviation or number (%).

### Contour sharpness

#### AIDR 3D versus FBP

**Fig 4:**
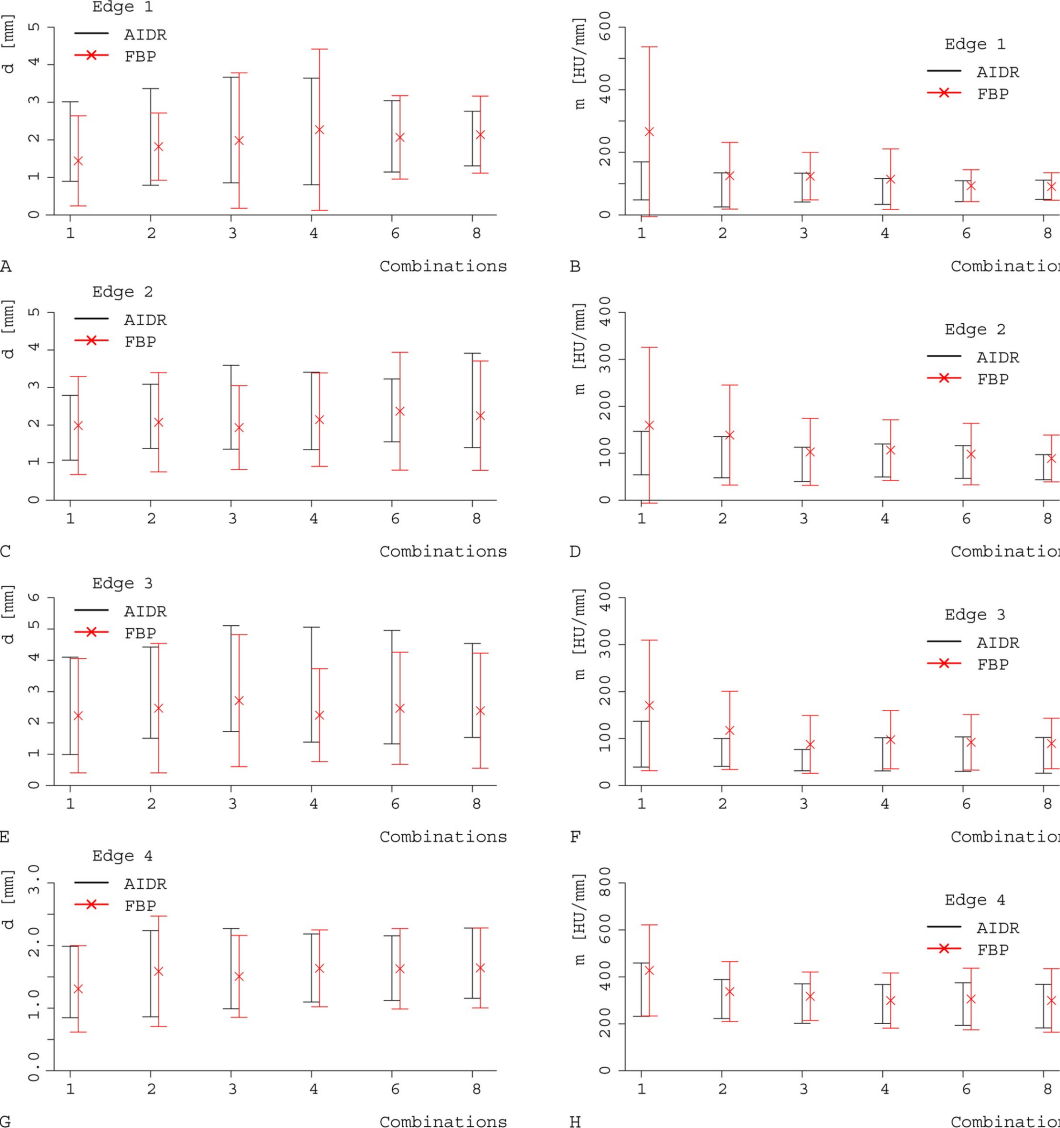
Comparison of the contour sharpness parameters. Values are given as mean ±SD between AIDR 3D (black) and FBP (red) and for the different levels of temporal averaging (1–8; x axis). We used two different quantitative parameters: the distance between 25%-75% of the maximal grey value (d; A, C, E, G) and the slope (m; B, D, F, H) at four different edges: edge 1 (A, B), edge 2 (C, D), edge 3 (E, F), and edge 4 (G, H). At all 4 edges, higher levels of temporal averaging and AIDR 3D showed poorer contour sharpness parameters (higher values for d, lower values for m) compared with FBP.

**Fig 5:**
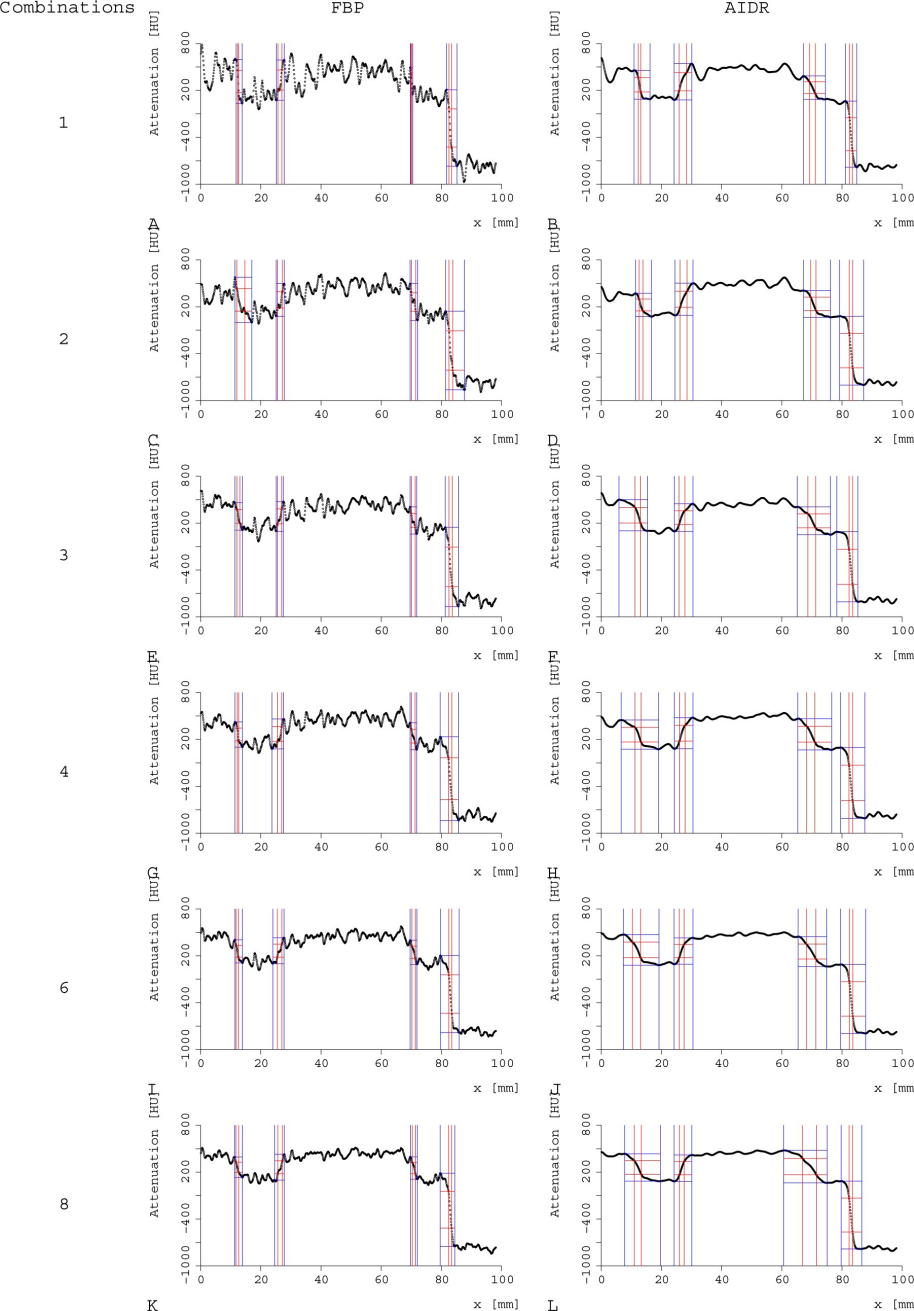
Demonstration of the special distribution of grey values across the 4 different edges measured. Comparison of FBP (A, C, E, G, I, K) and AIDR 3D strong levels (B, D, F, H, J, L) and of the different temporal averaging levels: no temporal averaging (A, B), combination of 2 3D datasets (C, D), combination of 3 3D datasets (E, F), combination of 4 3D datasets (G, H), combination of 6 3D datasets (I, J), and combination of 8 3D datasets (K, L).

**Fig 6:**
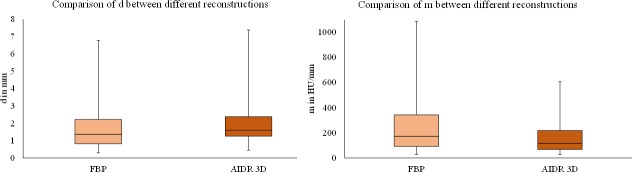
Boxplots of the comparison of contour sharpness parameters between FBP and iterative reconstruction. The comparison includes the distance between 25–75% of the maximal grey value (d; left diagram) and the slope in the contour (m; right diagram) between the two reconstructions—FBP and AIDR 3D strong levels. AIDR 3D was characterised by lower contour sharpness parameters, as indicated by higher values for the difference (d) and lower values for the slope (m).

Contour sharpness parameters were slightly inferior for AIDR 3D strong versus FBP at all 4 edges of the myocardium, as demonstrated by both methods: (d) higher values for the distance between 25% and 75% of the maximal grey value, and (m) lower values for the slope between 25% and 75% (Figs 4–6; S1 and S2 Tables). Note the visual impression of the effect of smoothening of edges with a less noisy appearance and less variation in Hounsfield units (HU) for AIDR 3D.

In detail, the comparison of the slope between the point of 25% and 75% (m) showed higher values for FBP than for strong levels of AIDR 3D (FBP 255.9±123.9 HU/mm versus AIDR 3D 160.6±123.5 HU/mm, p<0.023; Table 2). The distance between 25% and 75% of the maximal grey value (d) tended to be higher for AIDR 3D strong compared with FBP, but the difference was not statistically significant (FBP 1.7±0.4 mm versus AIDR 3D 2.0±0.5 mm, p>0.059; Table 2).

**Table 2:** Comparison of m and d between different reconstructions and levels of temporal averaging.

m	HU/ mm	Edge 1		Edge 2		Edge 3		Edge 4		mean	
TA	1	266.0	(271.2)	159.7	(165.8)	170.6	(139.1)	427.4	(194.1)	218.3	(67.5)
	2	125.3	(106.4)	138.7	(106.4)	117.3	(83.3)	337.2	(127.6)	121.3	(5.7)
	3	123.8	(75.8)	102.8	(71.3)	87.6	(61.6)	317.4	(103.4)	105.7	(25.6)
	4	114.3	(96.8)	106.8	(64.6)	97.6	(62.1)	299.3	(117.5)	105.9	(11.8)
	6	93.4	(51.1)	98.2	(65.4)	92.0	(59.2)	305.8	(131.3)	92.7	(1.0)
	8	90.7	(44.0)	88.9	(49.7)	89.5	(53.7)	299.5	(135.2)	90.1	(0.9)
	*P*	*<0*.001*^1^		*0*.010*^2^		*<0*.001*^3^		*0*.003*^4^			
Reco	AIDR	108.7	(60.8)	100.4	(46.2)	88.0	(48.8)	345.5	(113.7)	98.3	(14.6)
	FBP	266.0	(271.2)	159.7	(165.8)	170.6	(139.1)	427.4	(194.1)	218.3	(67.5)
	*P*	<0.001		0.023		<0.001		0.011			
**d**	mm										
TA	1	1.4	(1.2)	2.0	(1.3)	2.2	(1.8)	1.3	(0.7)	1.8	(0.6)
	2	1.8	(0.9)	2.1	(1.3)	2.5	(2.1)	1.6	(0.9)	2.1	(0.5)
	3	2.0	(1.8)	1.9	(1.1)	2.7	(2.1)	1.5	(0.7)	2.3	(0.5)
	4	2.3	(2.1)	2.1	(1.2)	2.2	(1.5)	1.6	(0.6)	2.3	(0.0)
	6	2.1	(1.1)	2.4	(1.6)	2.5	(1.8)	1.6	(0.6)	2.3	(0.3)
	8	2.1	(1.0)	2.2	(1.5)	2.4	(1.8)	1.6	(0.6)	2.3	(0.2)
	*p*	*0*.*105*		*0*.*680*		*0*.*665*		*0*.*052*			
Reco	AIDR	2.0	(1.1)	1.9	(0.9)	2.5	(1.6)	1.4	(0.6)	2.2	(0.4)
	FBP	1.4	(1.2)	2.0	(1.3)	2.2	(1.8)	1.3	(0.7)	1.8	(0.6)
	*p*	0.059		0.850		0.069		0.230			

*^1^ p for the single comparisons: 0–1: 0.001, 0–2: 0.044, 0–3: 0.007, 0–5: <0.001, 0–7: <0.001, 2–7: 0.024; for the remaining single comparisons: p n.s.

*^2^ p for the single comparisons: 0–2: 0.023, 1–7: 0.045; for the remaining single comparisons: p n.s.

*^3^ p for the single comparisons: 0–2: 0.003, 0–3: 0.027, 0–5: 0.019, 0–7: <0.001; for the remaining single comparisons: p n.s.

*^4^ p for the single comparisons: 0–2: 0.015, 0–3: <0.001, 0–5: 0.001, 0–7: 0.003; for the remaining single comparisons: p n.s

This was confirmed by the visual impression of a smooth edge, which was characterised by less variation in HU at a single edge localisation, as shown for AIDR 3D in Fig 2A versus FBP in Fig 2C.

Values are given as means (standard deviations). We used two different parameters for the quantitative evaluation of the contour sharpness: the distance between 25% and 75% of the maximal grey value (d) and the slope in the contour (m).

Comparisons were performed between FBP and strong levels of AIDR 3D (no temporal averaging; Reco = reconstruction), as well as between the different levels of temporal averaging (TA; no temporal averaging, combination of two, three, four, six and eight original 3D datasets from consecutive heart beats) for FBP reconstruction. Measurements were performed at 4 different edges of the myocardium.

Contour sharpness parameters were slightly poorer for AIDR 3D strong versus FBP at all 4 edges of the myocardium and also for higher levels of temporal averaging, as demonstrated by lower values for the slope (m; FBP versus AIDR 3D strong p<0.023 and temporal averaging p<0.01). The distance between 25% and 75% of the maximal grey value tended to be slightly increased, but the difference was not significant (p>0.059).

#### Different additions

**Fig 7:**
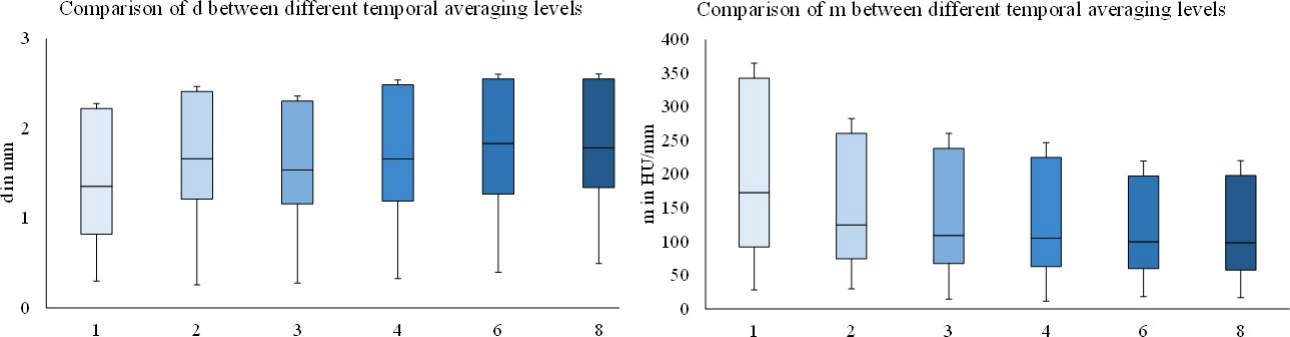
Boxplots of the comparison of contour sharpness parameters between different levels of temporal averaging. Comparisons of the distance between 25–75% of the maximal grey value (d; left diagram) and the slope in the contour (m; right diagram) were performed between the different temporal averaging levels (1 dataset and combination of 2, 3, 4, 6 and 8 datasets). Higher levels of temporal averaging showed reduced contour sharpness parameters, specifically higher values for the distance (d) and lower values for the slope (m).

Higher levels of temporal averaging led to slightly poorer values for both contour sharpness parameters, demonstrated by higher values for the distance between 25% and 75% of the maximal grey value and lower values for the slope (Figs 4, 5 and 7, Table 2, S1 and S2 Tables).

Thus, with increasing combination levels, the slope in the contour was poorer at all 4 edges (p<0.01 at all 4 edges; m for maximal temporal averaging 142.2±104.9 HU/mm versus 255.9±123.9 HU/mm without temporal averaging). The distance between 25% and 75% showed the same tendency, but without statistical significance (p>0.052 at all 4 edges). The best (lowest) values were achieved without temporal averaging, the maximal temporal averaging levels showed the highest values (d was 1.7±0.4 mm without temporal averaging versus 2.1±0.3 mm with maximal addition level), indicating the strongest reduction of contour sharpness. This goes in line with the visual impression of a smooth edge which was characterised by less variation in HU at a single edge localisation with increasing levels of temporal averaging (AIDR 3D Fig 2B, FBP Fig 2D).

## Discussion

Both noise reduction methods investigated here, iterative reconstruction and temporal averaging, degraded contour sharpness. In our analysis, both objective contour sharpness parameters deteriorated for strong levels of AIDR 3D compared to FBP and for increasing levels of temporal averaging. The use of two different but complementary parameters for contour sharpness analysis is in agreement with established approaches that have been optimised for the analysis of myocardial contours [21]. These quantitative image quality parameters were measured in 4 representative anatomical locations for myocardial CT perfusion imaging and thus included all available edges of the left-ventricular myocardium.

Since poorer contour sharpness leads to blurring and thus reduces the evaluability of the affected structures, contour sharpness is supposed to be a very valuable quantitative parameter of image quality. Morphological delineation is needed for generating precise perfusion maps. Because accurate and sharp contours aid the segmentation, contour sharpness is an important quantitative parameter for dynamic CT perfusion imaging.

Published data to prove the feasibility of 4D CTP of the entire myocardium are still limited. A study conducted in 32 Asian patients [17] shows a good correlation of the myocardial blood flow and the coronary flow reserve determined by 4D CTP compared with the findings of positron emission tomography. We investigated additional objective (noise, SNR and CNR) and subjective image quality parameters [18], which have already been published in the main manuscript of our study. For 4D dynamic CTP, we chose a protocol aimed at acquiring 20 heart beats, which was not always possible in patients with low or high heart rate. This resulted in a 3-fold higher dose for the complete CTP compared with the CTA, even though the dose for each individual acquisition was relatively low (9±2 mSv for CTP versus 3±2 mSv for CTA). Scanning at several time points has different advantages: it reduces the risk of missing the optimal time point for identifying a perfusion defect and allows the calculation of the absolute myocardial blood flow, which is not possible with acquisition at a single point in time. Despite these advantages, finding the best balance between low radiation dose and optimal image quality is a huge challenge for myocardial 4D CTP. Different approaches exist and have been applied in our study to optimise image quality adjusting scanning parameters, which would have resulted in higher radiation exposure: strong levels of AIDR 3D and temporal averaging of consecutive heart beats. The AIDR 3D reconstruction algorithm uses both a scanner and a statistical noise model in combination with a projection noise estimation in the raw data domain, enabling the reduction of quantum and electronic noise [22]. Subsequently, the initial reference image is produced and incorporated into the iteration cycle, which is model-based and considers the anatomical region, including contours and edges, and the reconstruction kernel. At each iteration stage, the output image is compared with the reference image and special attention is paid to the contour, which can be reconstructed from the initial FBP image (so-called blending). With this approach, edges are supposed to be preserved in the smoothing cycles, and hence there should be no relevant degradation of image sharpness. In our analysis, contour sharpness was slightly reduced, suggesting that the AIDR 3D algorithm prioritises overall noise reduction over edge preservation. Nevertheless, this effect was not very strong. A recent study of Feger et al. [19] analysed the influence of AIDR 3D levels on contour sharpness parameters for CTA and did not find a relevant difference between AIDR 3D and FBP. This could be due to the different characteristics of coronary arteries and the myocardium. During CTA, the coronary arteries are very well contrasted, resulting in relatively sharp edges in general, whereas the myocardium is usually less well contrasted in CTP. Another important difference is that different scanning parameters are used for CTA and CTP. Since 4D myocardial CTP is acquired at multiple time points, scanning parameters for each single acquisition are reduced to meet the radiation exposure requirements for the examination of patients, resulting in higher image noise of the FBP image compared with the CTA acquisition. This could have an effect on the contour sharpness of FBP images and might explain differences in contour sharpness when iterative reconstruction is applied. In addition, we used temporal averaging by combining and overlapping different 3D CTP datasets acquired during consecutive heartbeats. Different temporal averaging levels are achieved by changing the number of 3D datasets incorporated to generate the image. Since the overlapped images are acquired at different time points, the resulting 3D dataset is expected to highlight perfusion abnormalities compared with the single images, because it is more likely to incorporate the optimal time point for detecting the deficit. In general, the optimal time point for delineating a perfusion deficit is difficult to predict and is therefore easy to miss when only images from a single acquisition are available. However, temporal averaging is supposed to reduce contour sharpness due to the overlap of multiple possibly misaligned 3D datasets. In addition, this approach also includes less well contrasted images, which may reduce the slope of the edges, especially for the contour between the ventricle and myocardium. In our study, higher levels of temporal averaging also slightly reduced contour sharpness parameters. In addition, we used no motion correction. Thus, even if scanning conditions are optimal (no respiratory or body motion during the scan), cardiac deformation cannot be fully prevented, nor can slight scan time variations within the RR cycle from beat to beat. As a consequence, contours will become more blurred with increasing temporal averaging levels, resulting in reduced contour sharpness parameters. The influence of temporal averaging on other objective (including signal, noise, SNR and CNR) and subjective image quality parameters (subjective quality, motion and evaluability of perfusion defects) was already analysed in the main study, which showed that these parameters were best at medium temporal averaging levels (averaging of 3 consecutive 3D datasets). In the main study, 15 patients underwent the reference standard stress MRI, and diagnostic accuracy for native images was also improved by the combination of 3 3D datasets (67+11–14% with native images versus 73+10–14% with combinations of three 3D datasets). However, diagnostic performance of dynamic CTP compared with stress MRI was still moderate, even if temporal averaging was applied.

In general, the diagnostic accuracy of dynamic myocardial CTP can further be improved if the results of the coronary CTA are additionally considered. CTA is highly accurate in detecting significant coronary stenosis compared with ICA as the reference standard [1, 2]. Since both CTA and ICA are pure anatomical diagnostic tests, limitations exist in the prediction of the haemodynamic relevance, especially in patients with intermediate coronary stenosis or severe coronary calcification. In those patients, considering both functional and anatomic imaging might be beneficial. In our study, 18 patients underwent CTA/CTP and ICA, and the correlation between coronary stenosis and consecutive perfusion deficit in the region of the respective coronary artery was moderate (diagnostic accuracy 67%; 95% CI [0.44; 0.89]). This demonstrates that further refinements of acquisition, reconstruction, and postprocessing of dynamic CTP are still required.

An important advantage of myocardial CTP instead of different functional tests (e.g. stress MRI, SPECT MPI) is the option to perform the examination in the same session as the coronary CTA which enables immediate functional information and facilitates the implementation in clinical routine. Special benefits of dynamic versus static CTP include the possibility of calculating the absolute myocardial blood flow and acquiring time-attenuation curves [16].

A major limitation of our study is the small sample size of only 29 patients. Nevertheless, it is one of the largest whole-heart CT myocardial perfusion dataset studies to date. In addition, to statistically deal with the relatively small patient collective, we performed intraindividual comparisons and adapted the p value according to Bonferroni correction. In this analysis, we did not use post-processing motion correction, which would have improved the contour sharpness, especially for higher levels of temporal averaging. Even though we minimised motion during scans by very careful patient preparation, it is not possible to completely eliminate cardiac motion. In addition, in this analysis, we only used two objective parameters, which cannot cover all aspects of image quality. Therefore, we evaluated the results of this sub-study taking the additional objective and subjective image quality parameters discussed in the main manuscript into account. The comparison between CTA/CTP (all patients) and CTP/MRI (15 patients) is also published within the main study. The results of the main study and this sub-study need to be seen as a whole, since they are complementary. Our scanning parameters were relatively low at 103±8 kV and 148±21 mA, which is a reasonable approach in clinical practice in order to comply with the ALARA radiation safety principle. Such low scanning parameters have an influence on the performance and image quality of AIDR 3D and temporal averaging in general and on contour sharpness in particular. There are different approaches to deal with multiple datasets [23, 24]. It would be interesting to evaluate how they affect image quality parameters in 4D CTP of the myocardium. In addition, it would be interesting to compare the diagnostic performance of myocardial CTP with FFR or stress echocardiography, which was not part of our study protocol.

In conclusion, our results show that AIDR 3D and temporal averaging slightly reduce the contour sharpness of the myocardium. Thus, further developments in image processing are needed to optimise contour sharpness of 4D myocardial CTP.

## Supporting information

S1 Appendix TREND checklist. (PDF)

S1 Table Contour sharpness parameters for FBP reconstructions. We used two different parameters for the quantitative evaluation of the contour sharpness: the distance between 25% and 75% of the maximal grey value (d) and the slope in the contour (m). Comparisons were performed between the different levels of temporal averaging (TA; no temporal averaging, combination of two, three, four, six and eight original 3D datasets from consecutive heart beats). Measurements were performed at 4 representative edge localisations of the myocardium. Results were recorded for 3 different slice thicknesses.
(DOCX)

S2 Table Contour sharpness parameters for AIDR 3D reconstructions. We used two different parameters for the quantitative evaluation of the contour sharpness: the distance between 25% and 75% of the maximal grey value (d) and the slope in the contour (m). Comparisons were performed between the different levels of temporal averaging (TA; no temporal averaging, combination of two, three, four, six and eight original 3D datasets from consecutive heart beats). Measurements were performed at 4 representative edge localisations of the myocardium. Results were recorded for 3 different slice thicknesses.
(DOCX)

## Abbreviations

AIDR 3D: Adaptive Iterative Dose Reduction 3D
CTA: computed tomography angiography
CNR: contrast-to-noise ratio
CCA: conventional coronary angiography
CAD: coronary artery disease
d: distance between 25% and 75% of the maximal grey value
FOV: field of view
FBP: filtered back projection
LV: left ventricle
MRI: magnetic resonance imaging
MPR: multi-planar reconstruction
SNR: signal-to-noise ratio
SPECT-MPI: single-photon emission computed tomography myocardial perfusion imaging
m: slope between 25% and 75% of the maximal grey value
