# Supplementary material for: Effect of iterative reconstruction and temporal averaging on contour sharpness in dynamic myocardial CT perfusion: Sub-analysis of the prospective 4D CT perfusion pilot study
Source: PLoS One. 2018 Oct 16;13(10):e0205922. doi: 10.1371/journal.pone.0205922 (PMC6191149; doi:10.1371/journal.pone.0205922)
Supplement: S1 Table — We used two different parameters for the quantitative evaluation of the contour sharpness: the distance between 25% and 75% of the maximal grey value (d) and the slope in the contour (m). Comparisons were performed between the different levels of temporal averaging (TA; no temporal averaging, combination of two, three, four, six and eight original 3D datasets from consecutive heart beats). Measurements were performed at 4 representative edge localisations of the myocardium. Results were recorded for 3 different slice thicknesses. (DOCX) [file pone.0205922.s002.docx]

| **FBP** | Slice th. | TA | Edge 1 | | | | Edge 2 | | | | Edge 3 | | | | Edge 4 | | | | mean | | | |
| --- | --- | --- | --- | --- | --- | --- | --- | --- | --- | --- | --- | --- | --- | --- | --- | --- | --- | --- | --- | --- | --- | --- |
|  |  |  | m | | d | | m | | d | | m | | d | | m | | d | | m | | d | |
|  | 0mm | 0 | 266.0 | (271.2) | 1.4 | (1.2) | 159.7 | (165.8) | 2.0 | (1.3) | 170.6 | (139.1) | 2.2 | (1.8) | 427.4 | (194.1) | 1.3 | (0.7) | 255.9 | (123.9) | 1.7 | (0.4) |
|  |  | 1 | 125.3 | (106.4) | 1.8 | (0.9) | 138.7 | (106.4) | 2.1 | (1.3) | 117.3 | (83.3) | 2.5 | (2.1) | 337.2 | (127.6) | 1.6 | (0.9) | 179.6 | (105.4) | 2.0 | (0.4) |
|  |  | 2 | 123.8 | (75.8) | 2.0 | (1.8) | 102.8 | (71.3) | 1.9 | (1.1) | 87.6 | (61.6) | 2.7 | (2.1) | 317.4 | (103.4) | 1.5 | (0.7) | 157.9 | (107.4) | 2.0 | (0.5) |
|  |  | 3 | 114.3 | (96.8) | 2.3 | (2.1) | 106.8 | (64.6) | 2.1 | (1.2) | 97.6 | (62.1) | 2.2 | (1.5) | 299.3 | (117.5) | 1.6 | (0.6) | 154.5 | (96.8) | 2.1 | (0.3) |
|  |  | 5 | 93.4 | (51.1) | 2.1 | (1.1) | 98.2 | (65.4) | 2.4 | (1.6) | 92.0 | (59.2) | 2.5 | (1.8) | 305.8 | (131.3) | 1.6 | (0.6) | 147.4 | (105.7) | 2.1 | (0.4) |
|  |  | 7 | 90.7 | (44.0) | 2.1 | (1.0) | 88.9 | (49.7) | 2.2 | (1.5) | 89.5 | (53.7) | 2.4 | (1.8) | 299.5 | (135.2) | 1.6 | (0.6) | 142.2 | (104.9) | 2.1 | (0.3) |
|  | 5mm | 0 | 136.3 | (100.7) | 1.9 | (1.3) | 125.8 | (89.1) | 2.1 | (1.2) | 87.7 | (57.8) | 2.7 | (2.0) | 344.9 | (134.7) | 1.5 | (0.6) | 173.7 | (116.0) | 2.0 | (0.5) |
|  |  | 1 | 99.2 | (75.7) | 2.1 | (1.9) | 123.4 | (81.5) | 1.9 | (1.0) | 74.2 | (41.1) | 2.9 | (2.1) | 311.7 | (83.0) | 1.5 | (0.6) | 152.1 | (108.2) | 2.1 | (0.6) |
|  |  | 2 | 101.6 | (66.2) | 2.1 | (1.3) | 96.2 | (57.6) | 2.1 | (1.7) | 63.1 | (42.2) | 3.1 | (1.6) | 286.0 | (79.6) | 1.7 | (0.7) | 136.7 | (101.0) | 2.3 | (0.6) |
|  |  | 3 | 89.1 | (57.8) | 2.0 | (1.3) | 85.8 | (46.7) | 2.4 | (1.0) | 63.5 | (36.4) | 3.2 | (1.6) | 283.7 | (84.4) | 1.7 | (0.6) | 130.6 | (102.7) | 2.3 | (0.7) |
|  |  | 5 | 86.3 | (39.7) | 1.8 | (1.0) | 81.6 | (46.1) | 2.5 | (1.1) | 57.0 | (26.3) | 3.3 | (1.5) | 279.9 | (95.0) | 1.7 | (0.7) | 126.2 | (103.2) | 2.3 | (0.7) |
|  |  | 7 | 83.5 | (35.8) | 2.0 | (0.8) | 72.7 | (35.0) | 2.5 | (1.3) | 48.7 | (18.9) | 3.4 | (1.6) | 273.2 | (98.0) | 1.8 | (0.7) | 119.5 | (103.5) | 2.4 | (0.7) |
|  | 8mm | 0 | 116.3 | (83.3) | 2.4 | (1.9) | 111.1 | (61.1) | 1.9 | (1.0) | 76.4 | (55.6) | 2.6 | (1.4) | 303.8 | (138.5) | 1.7 | (0.7) | 151.9 | (102.8) | 2.2 | (0.4) |
|  |  | 1 | 81.3 | (58.1) | 2.4 | (1.8) | 99.9 | (61.1) | 2.3 | (0.9) | 62.4 | (35.2) | 3.5 | (2.2) | 278.9 | (94.2) | 1.8 | (0.9) | 130.6 | (100.0) | 2.5 | (0.7) |
|  |  | 2 | 87.9 | (53.5) | 2.3 | (1.2) | 78.0 | (42.1) | 2.7 | (2.0) | 58.5 | (42.0) | 3.5 | (2.2) | 255.2 | (85.0) | 1.9 | (0.9) | 119.9 | (91.0) | 2.6 | (0.7) |
|  |  | 3 | 75.4 | (47.1) | 2.3 | (1.4) | 83.9 | (43.9) | 2.5 | (1.7) | 53.8 | (26.1) | 3.7 | (1.8) | 251.2 | (88.7) | 2.0 | (0.9) | 116.1 | (91.0) | 2.6 | (0.8) |
|  |  | 5 | 72.3 | (34.2) | 2.3 | (1.2) | 75.7 | (41.2) | 2.9 | (2.3) | 52.3 | (26.8) | 3.6 | (1.6) | 247.1 | (93.5) | 2.0 | (0.8) | 111.8 | (90.7) | 2.7 | (0.7) |
|  |  | 7 | 75.0 | (33.3) | 2.3 | (1.1) | 68.5 | (31.3) | 2.6 | (1.1) | 44.1 | (20.0) | 3.8 | (1.6) | 243.4 | (97.7) | 2.1 | (0.9) | 107.7 | (91.4) | 2.7 | (0.8) |

**S1 Table. Contour sharpness parameters for FBP reconstructions.**
