# Supplementary material for: Effect of iterative reconstruction and temporal averaging on contour sharpness in dynamic myocardial CT perfusion: Sub-analysis of the prospective 4D CT perfusion pilot study
Source: PLoS One. 2018 Oct 16;13(10):e0205922. doi: 10.1371/journal.pone.0205922 (PMC6191149; doi:10.1371/journal.pone.0205922)
Supplement: S2 Table — We used two different parameters for the quantitative evaluation of the contour sharpness: the distance between 25% and 75% of the maximal grey value (d) and the slope in the contour (m). Comparisons were performed between the different levels of temporal averaging (TA; no temporal averaging, combination of two, three, four, six and eight original 3D datasets from consecutive heart beats). Measurements were performed at 4 representative edge localisations of the myocardium. Results were recorded for 3 different slice thicknesses. (DOCX) [file pone.0205922.s003.docx]

**S2 Table. Contour sharpness parameters for AIDR 3D reconstructions.**

| **AIDR 3D** | Slice th. | TA | Edge 1 | | | | Edge 2 | | | | Edge 3 | | | | Edge 4 | | | | mean | | | |
| --- | --- | --- | --- | --- | --- | --- | --- | --- | --- | --- | --- | --- | --- | --- | --- | --- | --- | --- | --- | --- | --- | --- |
|  |  |  | m | | d | | m | | d | | m | | d | | m | | d | | m | | d | |
|  | 0mm | 0 | 108.7 | (60.8) | 2.0 | (1.1) | 100.4 | (46.2) | 1.9 | (0.9) | 88.0 | (48.8) | 2.5 | (1.6) | 345.5 | (113.7) | 1.4 | (0.6) | 160.6 | (123.5) | 2.0 | (0.5) |
|  |  | 1 | 80.1 | (54.5) | 2.1 | (1.3) | 91.7 | (43.9) | 2.2 | (0.9) | 70.4 | (29.7) | 3.0 | (1.5) | 305.6 | (83.0) | 1.6 | (0.7) | 137.0 | (112.8) | 2.2 | (0.6) |
|  |  | 2 | 87.5 | (46.1) | 2.3 | (1.4) | 76.4 | (36.5) | 2.5 | (1.1) | 54.0 | (22.6) | 3.4 | (1.7) | 286.3 | (84.1) | 1.6 | (0.6) | 126.0 | (107.7) | 2.4 | (0.7) |
|  |  | 3 | 75.0 | (41.4) | 2.2 | (1.4) | 84.7 | (35.1) | 2.4 | (1.0) | 66.5 | (35.4) | 3.2 | (1.8) | 284.2 | (83.1) | 1.6 | (0.5) | 127.6 | (104.7) | 2.4 | (0.7) |
|  |  | 5 | 75.9 | (33.5) | 2.1 | (1.0) | 81.4 | (34.8) | 2.4 | (0.8) | 66.8 | (36.8) | 3.1 | (1.8) | 284.2 | (90.8) | 1.6 | (0.5) | 127.1 | (105.0) | 2.3 | (0.6) |
|  |  | 7 | 80.4 | (31.0) | 2.0 | (0.7) | 70.4 | (26.8) | 2.7 | (1.3) | 64.2 | (38.1) | 3.0 | (1.5) | 275.2 | (92.6) | 1.7 | (0.6) | 122.5 | (102.0) | 2.4 | (0.6) |
|  | 5mm | 0 | 90.8 | (47.4) | 2.2 | (1.1) | 98.1 | (41.3) | 1.9 | (0.7) | 61.9 | (24.9) | 3.2 | (1.7) | 298.0 | (96.9) | 1.6 | (0.6) | 137.2 | (108.3) | 2.2 | (0.7) |
|  |  | 1 | 73.3 | (50.6) | 2.4 | (1.7) | 89.8 | (40.7) | 2.2 | (0.8) | 52.1 | (17.7) | 3.8 | (1.7) | 279.8 | (71.0) | 1.6 | (0.6) | 123.8 | (105.2) | 2.5 | (0.9) |
|  |  | 2 | 84.0 | (47.7) | 2.1 | (1.1) | 76.3 | (44.5) | 2.6 | (1.2) | 50.3 | (31.8) | 3.9 | (1.5) | 262.0 | (72.1) | 1.8 | (0.6) | 118.2 | (97.0) | 2.6 | (0.9) |
|  |  | 3 | 68.4 | (36.5) | 2.2 | (1.2) | 78.5 | (33.6) | 2.6 | (1.2) | 52.1 | (22.1) | 4.1 | (1.8) | 260.2 | (76.7) | 1.8 | (0.7) | 114.8 | (97.6) | 2.7 | (1.0) |
|  |  | 5 | 72.3 | (29.6) | 2.1 | (1.0) | 74.5 | (30.8) | 2.7 | (1.3) | 53.2 | (26.3) | 3.7 | (1.5) | 258.5 | (80.7) | 1.8 | (0.6) | 114.6 | (96.4) | 2.6 | (0.8) |
|  |  | 7 | 76.7 | (27.5) | 2.1 | (0.8) | 66.0 | (23.1) | 2.8 | (1.3) | 51.4 | (34.1) | 3.8 | (1.6) | 251.2 | (84.5) | 1.9 | (0.7) | 111.3 | (93.8) | 2.6 | (0.8) |
|  | 8mm | 0 | 85.4 | (42.0) | 2.2 | (1.0) | 88.2 | (36.4) | 2.2 | (1.0) | 58.9 | (27.6) | 3.2 | (1.8) | 266.3 | (96.5) | 1.8 | (0.7) | 124.7 | (95.3) | 2.3 | (0.6) |
|  |  | 1 | 68.7 | (44.3) | 2.3 | (1.2) | 82.9 | (37.6) | 2.5 | (0.9) | 51.5 | (26.3) | 3.9 | (1.9) | 255.8 | (74.1) | 1.8 | (0.7) | 114.7 | (94.9) | 2.6 | (0.9) |
|  |  | 2 | 76.2 | (39.9) | 2.3 | (1.1) | 68.4 | (32.5) | 2.9 | (1.6) | 50.6 | (34.3) | 3.9 | (1.7) | 238.0 | (69.3) | 1.9 | (0.7) | 108.3 | (87.1) | 2.7 | (0.8) |
|  |  | 3 | 63.0 | (33.6) | 2.4 | (1.1) | 74.3 | (32.1) | 2.8 | (1.3) | 52.2 | (30.4) | 3.9 | (1.5) | 239.0 | (72.3) | 1.9 | (0.7) | 107.1 | (88.4) | 2.7 | (0.8) |
|  |  | 5 | 66.5 | (27.0) | 2.3 | (1.0) | 69.9 | (29.0) | 2.8 | (1.3) | 49.9 | (25.1) | 3.8 | (1.5) | 235.1 | (78.2) | 2.0 | (0.7) | 105.4 | (86.9) | 2.7 | (0.8) |
|  |  | 7 | 70.7 | (25.6) | 2.3 | (0.9) | 62.7 | (21.6) | 2.9 | (1.3) | 50.6 | (40.0) | 4.2 | (1.4) | 227.1 | (86.2) | 2.3 | (1.3) | 102.8 | (83.3) | 2.9 | (0.9) |
